# Recruiting people with selected citizenships for the health interview survey GEDA Fokus throughout Germany: evaluation of recruitment efforts and recommendations for future research

**DOI:** 10.1186/s12874-024-02328-w

**Published:** 2024-09-12

**Authors:** Carmen Koschollek, Beate Gaertner, Julia Geerlings, Ronny Kuhnert, Elvira Mauz, Claudia Hövener

**Affiliations:** 1 Department for Epidemiology and Health Monitoring, Robert Koch Institute, General-Pape-Straße 62-66, 12101 Berlin, Germany; 2 DZHK (German Centre for Cardiovascular Research), partner site Berlin, Berlin, Germany

**Keywords:** Migration, Survey research methods, Hard to survey, Mixed-mode, Multilingual, Sequential design, Population-based, Random sample

## Abstract

**Background:**

Germany is the second most common country of immigration after the US. However, people with own or familial history of migration are not represented proportionately to the population within public health monitoring and reporting. To bridge this data gap and enable differentiated analyses on migration and health, we conducted the health interview survey GEDA Fokus among adults with Croatian, Italian, Polish, Syrian, or Turkish citizenship living throughout Germany. The aim of this paper is to evaluate the effects of recruitment efforts regarding participation and sample composition.

**Methods:**

Data collection for this cross-sectional and multilingual survey took place between 11/2021 and 5/2022 utilizing a sequential mixed-mode design, including self-administered web- and paper-based questionnaires as well as face-to-face and telephone interviews. The gross sample (*n* = 33436; age range 18–79 years) was randomly drawn from the residents’ registers in 120 primary sampling units based on citizenship. Outcome rates according to the American Association for Public Opinion Research, the sample composition throughout the multistage recruitment process, utilization of survey modes, and questionnaire languages are presented.

**Results:**

Overall, 6038 persons participated, which corresponded to a response rate of 18.4% (range: 13.8% for Turkish citizenship to 23.9% for Syrian citizenship). Home visits accounted for the largest single increase in response. During recruitment, more female, older, as well as participants with lower levels of education and income took part in the survey. People with physical health problems and less favourable health behaviour more often took part in the survey at a later stage, while participants with symptoms of depression or anxiety more often participated early. Utilization of survey modes and questionnaire languages differed by sociodemographic and migration-related characteristics, e.g. participants aged 50 years and above more often used paper- than web-based questionnaires and those with a shorter duration of residence more often used a translated questionnaire.

**Conclusion:**

Multiple contact attempts, including home visits and different survey languages, as well as offering different modes of survey administration, increased response rates and most likely reduced non-response bias. In order to adequately represent and include the diversifying population in public health monitoring, national public health institutes should tailor survey designs to meet the needs of different population groups considered *hard to survey* to enable their survey participation.

## Background

### Germany is a country of immigration

Throughout the last decades international migration has been rising, with 3.6% of the world’s population being migrants in 2022 [1]. Germany has been the second most common destination country after the United States since 2005 [2]. Of the 83 million people living in Germany in private households in 2022, 18.3% were born outside of Germany and 14.0% had no German citizenship [3]. Considering another definition of migration status, the proportion is even higher, such as *immigrants and their (direct) descendants* (24.3%) [4]. Hence, the representation of people subsumed within these categories in their heterogeneity in public health research surveys and reporting is essential. This means that population-based surveys should include people with a history of migration according to their proportion in the general population. However, to date, achieving this has been challenging throughout Europe [5, 6], including in Germany.

### Underrepresentation of people with a history of migration in population-based survey research in Germany

Population-based health surveys are regularly conducted by the Robert Koch Institute (RKI), Germany’s national public health institute, to fulfil its mandate of public health monitoring and reporting. Besides a constantly decreasing overall response rate faced by survey research in general [7], the inclusion of people with a history of migration proportionately to the population is challenging [8]. Within the regularly conducted health interview surveys *German Health Update (GEDA)* in particular, an underrepresentation is observed when considering either the country of birth or citizenship [8], which is probably attributable to the fact that the GEDA surveys are first and foremost conducted as telephone interviews and in the German language only.

Other population-based health or social surveys from Germany show the same pattern: within the baseline survey of the German *National Cohort* (03/2014–09/2019), a health examination survey among adults conducted in 18 study centres throughout Germany, the proportion of participants born abroad was 10.4% [9]. In the RKI-SOEP-II study, collecting data focusing on the SARS-CoV-2 pandemic via web- and paper-based questionnaires (11/2021–02/2022), utilizing the *German Socioeconomic Panel*, only 10.7% of adults were born abroad (unweighted, own calculations) [10]. And in the last *German General Social Survey (ALLBUS)* from 2021, collecting data via web- and paper-based questionnaires (06/21–08/21), the proportion of participants born abroad was 11.1% (own calculations) [11]. All these surveys used samples from population registers, hence, all population groups registered should be represented. So according to Tourangeau [12], the challenges in including people with a history of migration in population-based surveys in these cases cannot be ascribed to them being ‘*hard-to-sample*’ (p. 3), ‘*hard-to-identify*’ (p. 6), or ‘*hard-to-reach*’ (p. 10), but probably specifically lie in ‘*persuading’* (p. 12) and ‘*interviewing’* (p. 14) them.

### Approaches for motivating people for survey participation

Some researchers suggest that challenges in motivating sample persons for survey participation are related to their involvement in their social environment, i.e. being less involved in their communities [13, 14]. Hence, specific recruitment efforts might be necessary to reduce non-response bias among those less involved, e.g. offering incentives [13]. Furthermore, other researchers express the need to actively involve the population groups or communities addressed, for example, in survey planning or the design of questionnaires and survey materials, and to involve community members as interviewers [15]. However, there are few examples that have involved the communities under study on a national level [16]. This is easier to implement in small-scale surveys including different cities or regions or using non-random samples – for example, participatory health research [17]. However, establishing personal contact through home visits in order to build trust might bridge such gaps in population-based surveys on a national level [14, 18–22]. In addition, many other possible solutions to overcome the challenge of interviewing sample persons have also been published – for example, offering different modes of survey administration [13], offering proxy interviews or translated study materials and questionnaires [12].

In 2016, the project *Improving Health Monitoring in Migrant Populations (IMIRA)* was initiated at RKI to address the better inclusion of people with a history of migration in public health monitoring and reporting. We conducted a feasibility study in two German federal states, Berlin and Brandenburg, to test strategies to improve the inclusion of selected migrant groups within health interview surveys [23, 24]. The questionnaire included new concepts that were cognitively pre-tested, for example, sense of belonging and self-reported discrimination. Questionnaires were translated into Arabic, Croatian, Polish, Romanian and Turkish using a team translation approach [8]. In addition, focus group discussions with migrant representatives and migrant interviewers were conducted to evaluate study materials and to learn from their experiences during recruitment. The lessons learned in terms of promising recruitment strategies and feasibility of new questionnaire concepts [8] were implemented in the health interview survey GEDA Fokus [25] in 2021–2022 on a national level among people with Croatian, Italian, Polish, Syrian or Turkish citizenship (the only reliably captured migration-related characteristic within population registers [26]^1^), representing some of the major groups of people with a history of migration in Germany. The selection of these groups followed model calculations considering the stock as well as dynamics (inward and outward migration). The model calculations are described in more detail in the study protocol [25].

The aim of this paper is to evaluate the efforts undertaken to motivate sample persons for survey participation. The results on final disposition codes and outcome rates according to the *American Association for Public Opinion Research (AAPOR)* [27] will be presented as well as the results on the development of the sample composition throughout the recruitment process. Furthermore, the outcome of the utilization of different modes of survey administration and questionnaire languages will be presented.

## Methods

### Study design and sampling procedures

GEDA Fokus is a cross-sectional, multilingual health interview survey conducted from November 2021 to Mai 2022 by the RKI, Berlin, Germany to provide comprehensive health data on specific migrant groups in Germany. The target population comprised permanent residents of Germany aged between 18 and 79 years having a Croatian, Italian, Polish, Syrian, or Turkish citizenship. People with insufficient knowledge of German or one of the offered translation languages (Arabic, Croatian, Italian, Polish, and Turkish) and those who were not able to provide informed consent for participation were excluded from the study [25].

The study was funded by the German Federal Ministry of Health (Grant Number: ZMVI1-2518FSB411) and was approved without concern by the local ethics committee at Charité – Universitätsmedizin Berlin (EA1/250/21) and by the Commissioner for Data Protection of the RKI. Participation was voluntary; informed consent was provided [25].

A two-stage stratified cluster-sampling was applied. During the first stage, 120 primary sampling units (PSUs) were randomly selected from all municipalities in Germany by the GESIS – Leibnitz Institute for the Social Science, Mannheim, Germany. The random selection of PSUs was stratified based on the proportion of the population without German citizenship within districts (among others, see [25]) and the BIK-10 classification, a regional classification system for Germany, with higher classification figures indicating a larger size of the respective municipality [28]. During the second stage, sample persons between 18 and 79 years of age were randomly selected out of the registers of residents’ registration offices in the selected PSUs, based on their citizenship (first, second, or third^2^; Croatian, Italian, Polish, Syrian, or Turkish). Based on calculations on anticipated response rates [25] the number of persons to be drawn out of the registers differed by citizenship, but also by BIK classification, as it was expected that people with selected citizenships would predominantly live in larger cities. The gross sample comprised 33,436 persons. Further details are described elsewhere [25].

### Recruitment of participants

Recruitment was conducted between November 2021 and May 2022 in two tranches, both covering four sequential contact phases using bilingual information materials and questionnaires^3^ in accordance to the respective citizenship:

#### Phase I:

Mailed written invitation letter including login details for self-administering a web-based questionnaire (saq-web), either in German language only or bilingual with German and the respective translation language, according to citizenship (e.g. German–Italian).

#### Phase II:

Mailed reminder letter three weeks after initial invitation, including the login details for saq-web and additionally a bilingual paper-based questionnaire for self-administration (saq-paper).

#### Phase III:

Mailed second reminder letter including login details for saq-web three weeks after first reminder. Additionally, in PSUs with a BIK classification ≥ 8^4^, a home visit was announced.

#### Phase IV:

Home visits were conducted in PSUs with a BIK classification ≥ 8, aiming to realize a computer-assisted personal (CAPI) or telephone interview (CATI). If no one was reached at a sample persons’ address, interviewers left a bilingual contact card through the letter box with contact information for questions or an appointment for an interview. At least four contact attempts on differing days from Monday until Saturday and daytimes from mornings until evenings were made before an address was defined as unknown eligibility.

Each letter included the contact information of the study team (hotline, e-mail address) for addressing questions or refusing participation. The target sample per citizenship was 1200 participants and home visits were stopped in the respective group when achieving this. All study participants received a voucher of ten euros after participation as an incentive.

### Definitions and indicators

**Disposition codes** according to AAPOR standards [27] were assigned to all sample persons at the end of each contact phase. Disposition codes are defined as follows:

#### Category 1:

Interview: (*I*) *Complete Interview*: ≥ 80% of applicable questions answered either in self-administration or interview (codes 1.1), (*P*) *Partial Interview*: < 80% and ≥ 30% of applicable questions answered (codes 1.2);

#### Category 2:

Eligible, non-interview: (*R*) *Refusal and breakoff*: e.g. refused participation or < 30% of applicable questions answered (codes 2.1), (*NC*) *Non-contact*: e.g. sample person unavailable during field period (codes 2.2), (*O*) *Other*,* non-refusals*: e.g. someone else answered the questionnaire (codes 2.3, 2.9);

#### Category 3:

Unknown eligibility, non-interview: (*UH*) *Unknown if household*: e.g. address could not be located (codes 3.1), (*UO*) *Unknown other*: miscellaneous reasons why nothing is known about address (codes 3.2–3.9);

#### Category 4:

Not eligible: (*NE*) *Not eligible*: e.g. sample person moved before field period (codes 4) [27].

All sample persons without final case closure were assigned to the temporary disposition code *UO* at the end of each contact phase, until they received their final disposition code when recruitment ended.

**Outcome rates** were calculated according to AAPOR standards [27]. We calculated the most conservative rates *Response Rate 1*, *Cooperation Rate 1*, and *Contact Rate 1*.

Study participants were defined as **early participants** if they participated in contact phase I after the invitation letter, as **intermediate participants** if they took part in contact phases II and III (first or second reminder), and as **late participants** if they participated in phase IV after home visits started.

The sample composition throughout the recruitment process is described based on **register-based data** (gender, age groups, citizenship, BIK classification of PSU) and on **self-reported data** in terms of *socio-economic* (educational level, equivalized disposable household income) and *migration-related characteristics* (German language proficiency, duration of residence); additionally, indicators on *physical health* (self-perceived health, long-standing health problem, activity limitations), *health behaviour* (current smoking status, achievement of the World Health Organisation (WHO) recommendations on aerobic physical activity) and *mental health* (symptoms of depression, anxiety disorder) as well as *psychosocial determinants* of health (social support, sense of belonging to the society in Germany, and self-reported experiences of discrimination in everyday life) are presented. The operationalization of the respective indicators is described in detail in Table 1.

**Table 1:** Operationalization of indicators describing the sample composition throughout the recruitment process, GEDA Fokus, Germany, 2021–2022

Indicator	Operationalization	Categories	
Register-based data			
Gender	Gender designation according to the register of the residents’ registration office.	female male	
Age groups	Age was calculated based on the register entry on date of birth and the reference date of sample drawing (tranche 1: 09/17/2021; tranche 2: 10/13/2021).	18–35 years 36–50 years 51–65 years 66–79 years	
Citizenship	Register entry in first, second, or third citizenship, used for sampling.	Croatian Italian Polish Syrian Turkish	
BIK classification	BIK classification [28] of the PSUs, dichotomized in municipalities and smaller cities (BIK < 8) vs. bigger cities (BIK ≥ 8). A BIK classification of 8 is ascribed to the core areas of cities with 100,000 to 500,000 inhabitants, classifications 9 and 10 are ascribed to the core and surrounding areas of cities with ≥ 500,000 inhabitants. A BIK classification of 7 is ascribed to the surrounding areas of cities with 100,000 to 500,000 inhabitants. Classifications of 6 and below are ascribed to cities and municipalities with less than 100,000 inhabitants.	BIK < 8 BIK ≥ 8	
Self-reported data			
Socio-economic characteristics			
Educational level	Based on responses on educational and vocational qualifications and classified according to the *International Standard Classification of Education (ISCED 2011)* into ‘low’ (ISCED 1–2), ‘medium’ (ISCED 3–4) and ‘high’ (ISCED 5–8) [29].	low medium high	
Equivalized disposable net income	Based on responses on the household net income and the number and age of household members. Missing values were imputed using methods of regression analyses, including information on gender, age, household composition, educational level, occupational position, and regional information on unemployment and income tax of the respective PSU [30]. For analyses, income groups were categorized as ‘low’ (quintile 1), ‘medium’ (quintiles 2–4), and ‘high’ (quintile 5).	low medium high	
Migration-related characteristics			
German language proficiency	Defined by the question on native language and the subjective assessment of German language proficiency of those participants not indicating German as their native language.	native/ very good good/ moderate poor/ very poor	
Duration of residence	Based on country of birth and calculated as the difference of the year 2022 and the year of moving to Germany of those not born in Germany.	≤ 5 years 6–10 years 11–20 years ≥ 21 years since birth	
Physical health			
Self-perceived health	*Minimum European Health Module* [31], question 1: ‘How is your health in general? Is it…’	very good/ good moderate/ poor/ very poor	
Long-standing health problem	*Minimum European Health Module* [31], question 2: ‘Do you have any long-standing illness or health problem? This refers to illnesses or health problems that last or are expected to last at least 6 months.’	yes no	
Activity limitations	*Minimum European Health Module* [31], question 3: ‘To what extent have you been limited because of a health problem in activities people usually do? Would you say you have been…’	moderately/ severely limited not limited	
Health behaviour			
Current smoking status	Answers on currently smoking were summarized as ‘Yes’ (daily/ occasional smokers) and ‘No’ (those participants who never smoked or have given up smoking).	yes no	
Achievement of the WHO recommendations on aerobic physical activity	Assessed by the sum of minutes spent on leisure time physical activity and cycling reaching at least 150 min per week (‘Yes’) or not (‘No’) using the EHIS-PAQ (questions 4 and 5 for cycling; questions 6 and 7 for leisure time physical activity, respectively) [32].	yes no	
Mental health			
Symptoms of depression	Assessed using the 9-item version of the *Patient Health Questionnaire* (PHQ-9); answers were summarized and the score was dichotomized at a cut-off value of ≥ 10 indicating the presence of depressive symptoms (‘Yes’), and below their absence (‘No’) [33, 34]. Cases with ≥ one missing value were excluded.	yes no	
Symptoms of anxiety disorder	Assessed using the 7-item anxiety scale (GAD-7); dichotomizing the sum score at a cut-off value of ≥ 10 indicating the presence of symptoms of anxiety disorder (‘Yes’), and below their absence (‘No’) [35]. Cases with ≥ one missing value were excluded.	yes no	
Psychosocial determinants of health			
Social support	Assessed using the 3-item *Oslo Social Support Scale* (OSSS-3) and categorized as ‘low’ (sum-scores 3–8), ‘medium’ (9–11), and ‘high’ (12–14) [36].	low medium high	
Sense of belonging to the society in Germany	Captured by the question ‘How much do you feel you belong to the society in Germany?’ [37].	very strongly/ strongly partly/ barely/ not at all	
Experiences of discrimination in everyday life	Assessed by a 5-item adapted version of the *Everyday Discrimination Scale* [37]. Answers over all five items were coded as ‘rarely/ never’ if each single item was answered alike, and coded as ‘very often/ often/ sometimes’ when participants answered at least one item with sometimes or more often. Cases with > 2 missing values were excluded. Information on the possible reasons for discrimination were left out for the analysis at hand; hence, the focus is on overall, but not on migration-related discrimination.	very often/ often/ sometimes rarely/ never	

PSUs: primary sampling units; EHIS-PAQ: European Health Interview Survey – Physical Activity Questionnaire

The **utilization of questionnaire language** was assessed by asking ‘In which language did you answer the questionnaire?’ with the answer options per citizenship group (e.g. Italian) ‘Exclusively German’, ‘Predominantly German’, ‘Exclusively Italian’, ‘Predominantly Italian’ and ‘I used both languages equally’. Answers on the exclusive or predominant utilization of the respective translation languages (Arabic, Croatian, Italian, Polish and Turkish) were summarized to ‘Exclusively translation’ and ‘Predominantly translation’.

### Statistical analyses

Outcome rates were calculated at the end of each contact phase according to AAPOR standards [27]. For these calculations the following formulas, provided by AAPOR, were used [27]:

#### Response rate 1

I/ (I + P) + (R + NC + O) + (UH + UO).

#### Cooperation Rate 1

I/ (I + P) + R + O.

#### Contact rate 1

(I + P) + R + O / (I + P) + R + O + NC + (UH + UO).

With I = complete Interview, P = partial Interview, R = refusal and breakoff, NC = non-contact, O = other, non-refusals, UH = unknown if household and UO = unknown other.

P﻿roviding proportions and respective 95% confidence intervals (95% CIs), early, intermediate, and late participants as well as the final sample are described; differences between groups according to 95% CIs are only considered to be significant if 95% CIs do not overlap.

The utilization of the mode of survey administration and questionnaire language are descriptively displayed using bar charts stratified by selected characteristics. Chi^2^ tests were calculated to detect the significance level of potential differences. Statistical significance was defined at *p* < 0.05.

Cases with missing values were excluded in the respective analyses. All analyses were conducted using Stata/SE 17.0 (Stata Corp., College Station, TX, USA, 2017).

## Results

## Sample description

Of the gross sample (*n* = 33436) 539 cases were excluded due to ineligibility. Of the adjusted gross sample (*n* = 32897), 46.0% were female and the median age was 41 years. The majority lived in PSUs with a BIK classification ≥ 8 (86.0%). Most sample persons had Turkish citizenship (26.6%) followed by those with Croatian (20.7%), Italian (20.4%), Polish (16.9%), and Syrian citizenship (15.4%).

Overall, 6038 participants took part in the survey, of whom 49.4% were female and the median age was 39 years. There were 90.5% living in PSUs with a BIK classification ≥ 8. A detailed description of the study population stratified by citizenship can be found in the study protocol [25].

### Final disposition codes and outcome rates

The final response rate 1 was 18.4% overall, with an initial response rate of 6.0%; the final contact phase including home visits accounted for the largest single increase in the response rate (+ 6.8%) (Table 2). The lowest response rate throughout all contact phases was observed in the group with Turkish citizenship, while it was highest in the group with Syrian citizenship. The cooperation rate 1 was 29.6% overall, ranging from 20.5% in the group with Turkish citizenship to 48.2% in that with Syrian citizenship. Overall contact rate 1 was 62.0%, with the lowest in the group with Italian citizenship (48.5%) and the highest in the group with Polish citizenship (75.4%).

Response rates differed by BIK classification of the PSU only in contact phase IV. Home visits in PSUs with a BIK classification ≥ 8 accounted for an increase in the response rate 1 (+ 7.6%) as well as in the contact rate 1 (+ 47.3%).

**Table 2:** Final disposition codes and outcome rates by citizenship and BIK classification, GEDA Fokus, Germany, 2021–2022

		Final disposition codes								Outcome rates		
	Contact phase	1.1 I	1.2 P	2.1 R	2.2 NC	2.0, 2.3 O	3.1 UH	3.2-3.9 UO	4 NE	Response Rate 1	Cooperation Rate 1	Contact Rate 1
Overall (n = 33436)	I	1999	10	327	2	63	21	30,853	161	6.0%	83.3%	7.2%
	II	2626	20	553	8	256	21	29,777	175	7.9%	76.0%	10.4%
	III	3859	58	1148	15	1845	21	26,308	182	11.6%	55.8%	20.8%
	IV	6038	98	5579	838	8696	11,648	0	539	18.4%	29.6%	62.0%
**Citizenship**												
Croatian (n = 6962)	I	386	2	43	0	6	0	6485	40	5.6%	88.3%	6.3%
	II	495	3	96	2	40	0	6284	42	7.2%	78.1%	9.2%
	III	796	13	229	4	363	0	5512	45	11.5%	56.8%	20.3%
	IV	1223	21	1092	93	2220	2162	0	151	18.0%	26.8%	66.9%
Italian (n = 6780)	I	485	2	75	0	10	0	6174	34	7.2%	84.8%	8.5%
	II	647	4	128	0	71	0	5895	35	9.6%	76.1%	12.6%
	III	931	11	260	1	483	0	5058	36	13.8%	55.3%	25.0%
	IV	1205	19	551	23	1482	3440	0	60	17.9%	37.0%	48.5%
Polish (n = 5723)	I	345	1	19	0	7	0	5314	37	6.1%	92.7%	6.5%
	II	469	2	55	4	25	0	5127	41	8.3%	85.1%	9.7%
	III	695	7	155	5	401	0	4419	41	12.2%	55.2%	22.1%
	IV	1193	11	1132	340	1863	1030	0	154	21.4%	28.4%	75.4%
Syrian (n = 5082)	I	467	1	152	0	39	21	4391	11	9.2%	70.9%	13.0%
	II	571	3	178	0	66	21	4231	12	11.3%	69.8%	16.1%
	III	742	10	245	0	302	21	3750	12	14.6%	57.1%	25.6%
	IV	1209	20	379	2	901	2544	0	27	23.9%	48.2%	49.6%
Turkish (n = 8889)	I	316	4	38	2	1	0	8489	39	3.6%	88.0%	4.1%
	II	444	8	96	2	54	0	8240	45	5.0%	73.8%	6.8%
	III	695	17	259	5	296	0	7569	48	7.9%	54.9%	14.3%
	IV	1208	27	2425	380	2230	2472	0	147	13.8%	20.5%	67.4%
**BIK classification of PSUs**												
BIK < 8 (n = 4609)	I	247	1	80	1	12	1	4256	11	5.4%	72.6%	7.4%
	II	365	5	124	4	47	1	4050	13	7.9%	67.5%	11.8%
	III	507	6	165	4	312	1	3601	13	11.0%	51.2%	21.5%
	IV *	574	7	174	4	414	3,423	0	13	12.5%	49.1%	25.4%
BIK ≥ 8 (n = 28827)	I	1752	9	247	1	51	20	26,597	150	6.1%	85.1%	7.2%
	II	2261	15	429	4	209	20	25,727	162	7.9%	77.6%	10.2%
	III	3352	52	983	11	1553	20	22,687	169	11.7%	56.4%	20.7%
	IV	5464	91	5405	834	8282	8,225	0	526	19.3%	28.4%	68.0%

* Time after home visits had started in the PSUs with BIK classification ≥ 8 until the end of recruitment

I – Complete Interview; P – Partial interview; R – Refusal and breakoff; NC – Non-contact;

O – Other, non-refusals; UH – Unknown if household; UO – Unknown Other; NE – Not eligible

**Response Rate 1**: I/ (I + P) + (R + NC + O) + (UH + UO);

**Cooperation Rate 1**: I/ (I + P) + R + O;

**Contact Rate 1**: (I + P) + R + O / (I + P) + R + O + NC + (UH + UO) [27]

### Sample composition throughout the recruitment process

Females, older participants, and those with low educational and income levels more often were intermediate or late participants. During recruitment, more participants with good or moderate German language proficiency could be included as well as those with a longer duration of residence. The same holds true for participants with Polish and Turkish citizenship. Those with Syrian citizenship were least often intermediate and those with Italian citizenship were least often late participants. In the intermediate contact phases, more participants with less favourable physical health outcomes took part; in terms of health behaviour this also holds true for the last contact phase. Those with symptoms of depression or anxiety were less often intermediate or late participants, which can also be observed for those reporting low social support, a lower sense of belonging to the society in Germany, or those reporting experiences of discrimination in their everyday life (Table 3).

**Table 3:** Sample composition among participants (early, intermediate, late, all) by selected characteristics, GEDA Fokus, Germany, 2021–2022

	early participants				intermediate participants		late participants		all participants	
**Register-based data – socio-demographic characteristics**										
Gender	*n* = 1999				*n* = 1860		*n* = 2179		*n* = 6038	
Female	46.0		43.8–48.2		**50.8**	**48.5–53.0**	**51.4**	**49.3–53.5**	49.4	48.1–50.7
Age groups	*n* = 1999				*n* = 1860		*n* = 2179		*n* = 6038	
18–35 years	49.7		47.5–51.9		**34.9**	**32.8–37.1**	**38.4**	**36.3–40.4**	41.0	39.8–42.3
36–50 years	31.1		29.1–33.2		32.9	30.8–35.1	31.4	29.5–33.4	31.8	30.6–33.0
51–65 years	13.7		12.3–15.3		**21.7**	**19.9–23.7**	**20.7**	**19.0–22.4**	18.7	17.7–19.7
66–79 years	5.5		4.6–6.6		**10.5**	**9.2–12.0**	9.6	3.8–10.9	8.5	7.8–9.2
Citizenship	*n* = 1999				*n* = 1860		*n* = 2179		*n* = 6038	
Croatian	19.3		17.6–21.1		22.0	20.2–24.0	19.6	18.0–21.3	20.3	19.3–21.2
Italian	24.3		22.4–26.2		24.0	22.1–26.0	**12.6**	**11.2–14.0**	20.0	19.0–21.3
Polish	17.2		15.7–19.0		18.8	17.1–20.7	**22.9**	**21.1–24.7**	19.8	18.8–20.8
Syrian	23.4		21.6–25.3		**14.8**	**13.2–16.5**	21.4	19.8–23.2	20.0	19.0–21.1
Turkish	15.8		14.3–17.5		**20.4**	**18.6–22.3**	**23.5**	**21.8–25.4**	20.0	19.0–21.0
**Self-reported data – socio-economic characteristics**										
Educational level	*n* = 1997				*n* = 1850		*n* = 2159		*n* = 6006	
low	18.5		16.8–20.2		**23.2**	**21.4–25.2**	**41.9**	**39.9–44.0**	28.4	27.3–29.5
medium	38.4		36.3–40.6		40.7	38.4–42.9	34.3	32.3–36.4	37.6	36.4–38.9
high	43.1		41.0–45.3		**36.1**	**34.0–38.3**	**23.8**	**22.0–25.6**	34.0	32.8–35.2
Equivalized disposable household income	*n* = 1994				*n* = 1839		*n* = 2135		*n* = 5968	
low	15.7		14.2–17.4		16.3	14.7–18.1	**20.2**	**18.5–22.0**	17.5	16.6–18.5
medium	54.7		52.2–56.9		**59.5**	**57.3–61.8**	**64.3**	**62.3–66.3**	59.6	58.4–60.9
high	29.5		27.6–32.6		**24.1**	**22.2–26.2**	**15.5**	**14.0–17.1**	22.9	21.8–23.9
**Self-reported data – migration-related characteristics**										
German language proficiency	*n* = 1999				*n* = 1797		*n* = 2151		*n* = 5947	
native/ very good	47.8		45.6–50–0.0		45.0	42.7–47.3	**37.4**	**35.4–39.5**	43.2	42.0–44.5
good/ moderate	43.6		41.4–45.8		47.1	44.8–49.4	**52.2**	**50.1–54.3**	47.7	46.5–49.0
poor/ very poor	8.6		7.5–9.9		7.9	6.7–9.2	10.4	9.2–11.8	9.1	8.3–9.8
Duration of residence	*n* = 1994				*n* = 1838		*n* = 2119		*n* = 5951	
≤ 5 years	18.2		16.5–19.9		15.0	13.5–16.7	16.1	14.6–17.7	16.5	15.5–17.4
6–10 years	27.6		25.7–29.6		**20.1**	**18.3–22.0**	27.1	25.3–29.1	25.1	24.0–26.2
11–20 years	7.2		6.2–8.4		7.7	6.5–9.0	**9.7**	**8.5–11.0**	8.2	7.6–9.0
≥ 21 years	23.7		21.9–25.7		**36.5**	**34.3–38.7**	**30.9**	**29.0–32.9**	30.2	29.1–31.4
since birth	23.3		21.5–25.2		20.8	19.0–22.7	**16.2**	**14.7–17.8**	20.0	19.0–21.0
**Self-reported data – physical health**										
Self-perceived health	*n* = 1999				*n* = 1857		*n* = 2176		*n* = 6032	
moderate/ poor/ very poor	19.5		17.8–21.2		**25.9**	**24.0–28.0**	**25.1**	**23.4–27.0**	23.5	22.4–24.6
Long-standing health problem	*n* = 1998				*n* = 1852		*n* = 2169		*n* = 6019	
Yes	35.7		33.6–37.8		**40.7**	**38.4–42.9**	**30.2**	**28.3–32.2**	35.2	34.0–36.5
Activity limitations	*n* = 1999				*n* = 1840		*n* = 2164		*n* = 6003	
moderate/ severe	26.0		24.1–28.0		**32.6**	**30.5–34.8**	28.1	26.2–30.0	28.8	27.6–29.9
**Self-reported data – health behaviour**										
Currently smoking	*n* = 1999				*n* = 1860		*n* = 2175		*n* = 6034	
Yes	30.9		38.9–32.9		**29.8**	**27.8–32.0**	**34.9**	**32.9–36.9**	32.0	30.8–33.2
Physically active *	*n* = 1992				*n* = 1745		*n* = 2101		*n* = 5838	
No	60.3		58.1–62.4		**65.5**	**63.2–67.7**	**75.1**	**73.2–76–9**	67.2	66.0–68.4
**Self-reported data – mental health**										
Depressive symptoms	*n* = 1985				*n* = 1795		*n* = 2136		*n* = 5916	
Yes	25.4		23.6–27.4		**21.2**	**19.3–23.1**	**17.2**	**15.7–18.9**	21.2	20.2–22.2
Symptoms of anxiety disorder	*n* = 1990				*n* = 1805		*n* = 2136		*n* = 5931	
Yes	18.1		16.5–19.8		14.9	13.3–16.6	**12.4**	**11.0–13.9**	15.1	14.2–16.0
**Self-reported data – psychosocial determinants of health**										
Social support	*n* = 1992				*n* = 1836		*n* = 2151		*n* = 5979	
low	29.7		27.7–31.7		27.3	25.3–29.4	**21.8**	**20.1–23.6**	26.1	25.0–27.2
medium	50.7		48.5–52.9		50.4	48.2–52.7	52.4	50.2–54–5	51.2	50.0–52.5
high	19.6		17.9–21.4		22.3	20.4–24.2	**25.9**	**24.0–27.7**	22.7	21.6–23.8
Sense of belonging to the society in Germany	*n* = 1981				*n* = 1828		*n* = 2163		*n* = 5972	
partly/ barely/ not at all	45.3		43.1–56.9		**38.2**	**36.1–40.5**	**33.3**	**31.3–35.3**	38.8	37.6–40.4
Experiences of discrimination in everyday life	*n* = 1999				*n* = 1852		*n* = 2173		*n* = 6024	
very often/ often/ sometimes	49.4		47.2–51.6		**40.9**	**38.7–43.1**	**33.1**	**31.2–35–1**	40.9	39.7–42.2

* Achievement of the WHO recommendations for aerobic physical activity

CI: confidence interval

Significant differences between intermediate and/or late participants and early participants were assumed according to non-overlapping 95% confidence intervals and are indicated in bold

### Utilization of modes of survey administration

**Fig. 1:**
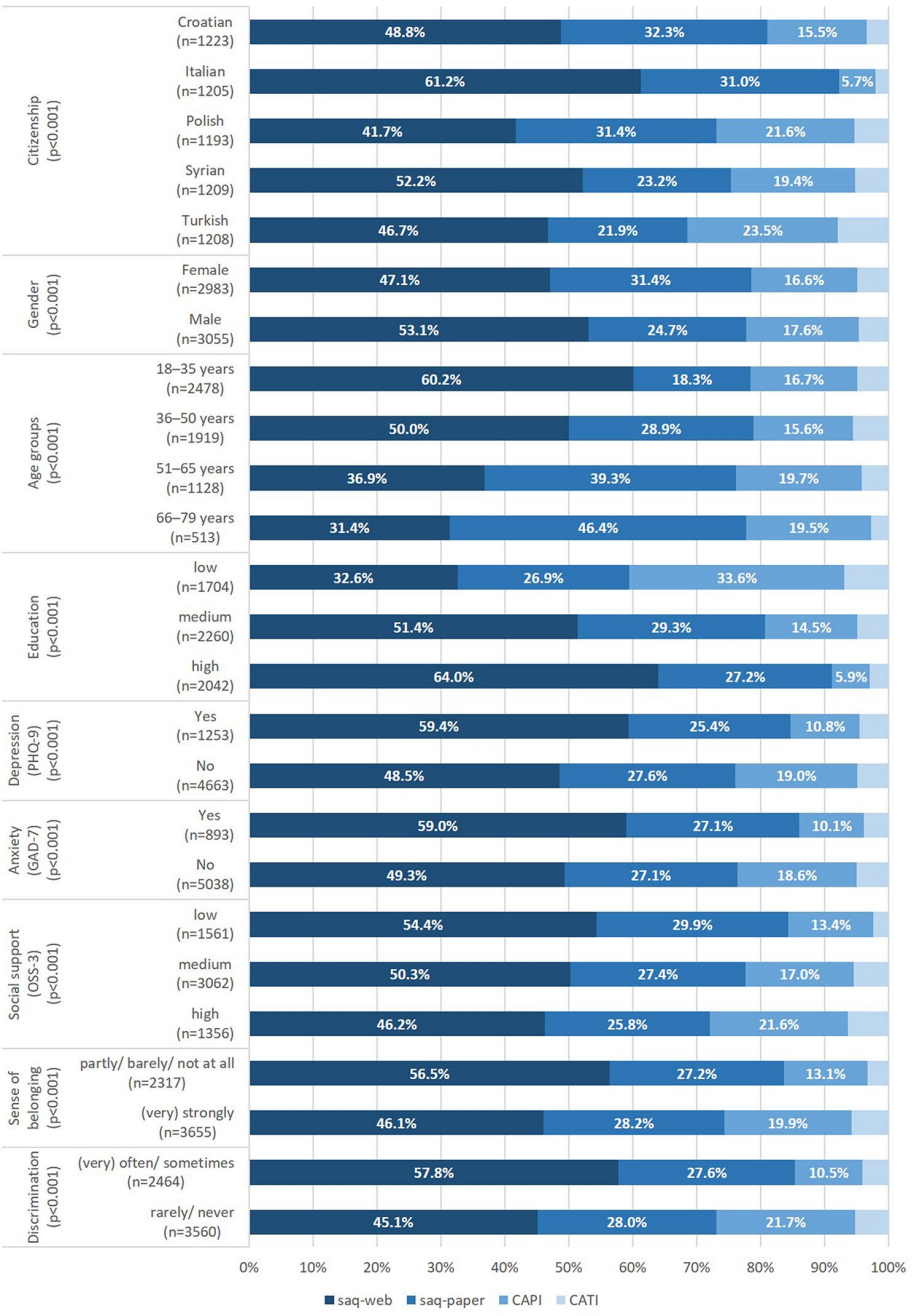
Modes of survey administration stratified by selected characteristics, GEDA Fokus, Germany, 2021–2022

The majority of participants utilized self-administered survey modes (saq-web: 50.2%; saq-paper: 28.0%), while every fifth participant had an interview (CAPI: 17.1%; CATI: 4.8%). Self-administration most often occurred in the group with Italian citizenship and least often in that with Turkish citizenship (Fig. 1). Male participants more often used saq-web, while females more often used saq-paper. Among participants below the age of 50, saq-web was most often chosen, while above the age of 50 saq-paper was preferred. The chosen mode of survey administration also varied by educational level: while participants with high education most often chose self-administration (91.2%), those with low education more often were interviewed (40.5%). Participants with symptoms of depression or anxiety as well as those with lower social support, a lower sense of belonging to the society in Germany and those with experiences of discrimination more often chose self-administration. All group differences were statistically significant (*p* < 0.001) according to chi²-tests.

### Utilization of questionnaire language

**Fig. 2:**
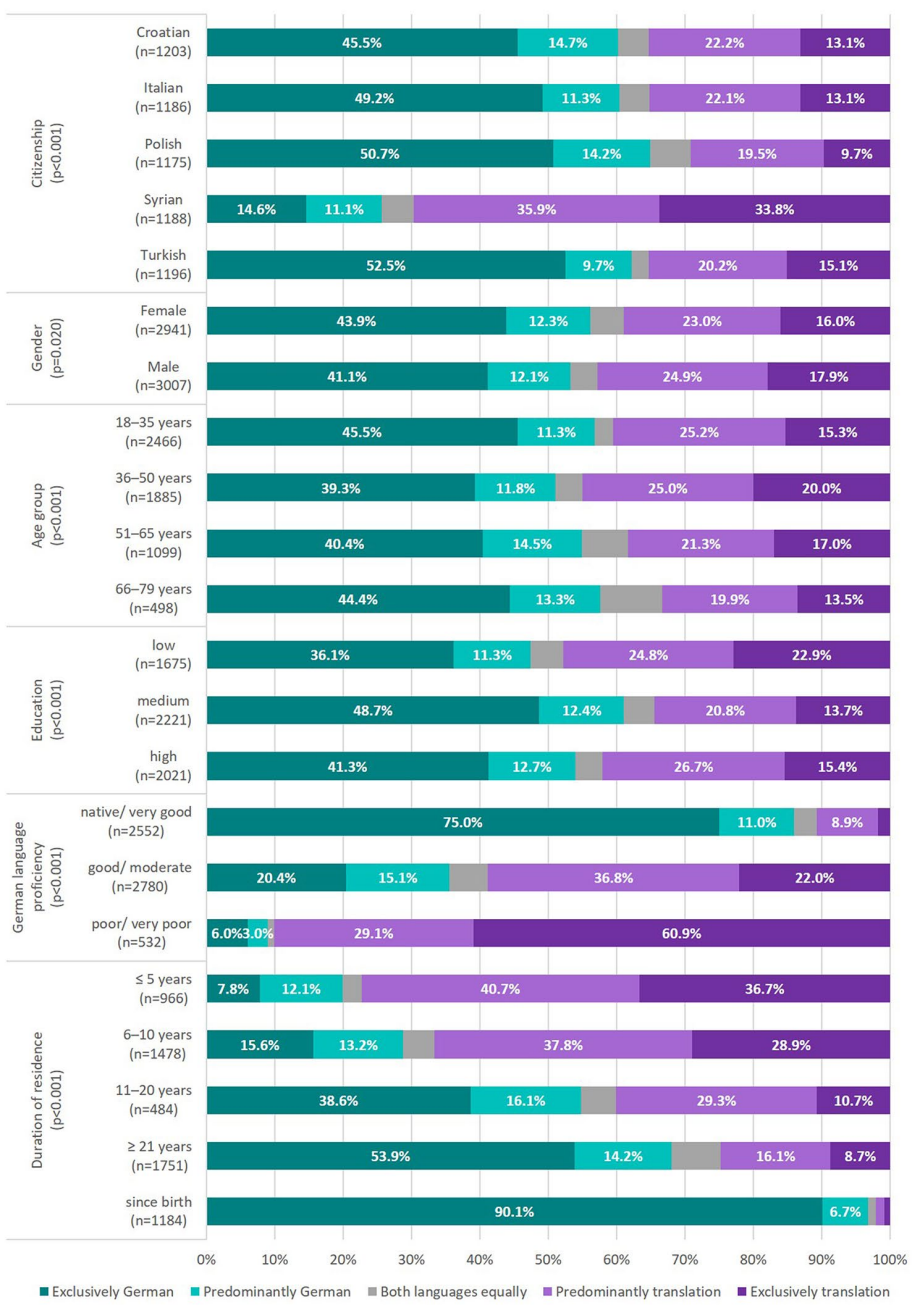
Utilization of questionnaire language stratified by selected characteristics, GEDA Fokus, Germany, 2021–2022

More than half of the participants (54.7%) answered the questionnaire exclusively or predominantly in German, while 40.9% exclusively or predominantly used the translation. German language was most often used by participants with Polish citizenship and least often by those with Syrian citizenship (Fig. 2). Females used German slightly more often compared to males. Participants aged between 36 and 50 years most often used the translation, while those with medium level of education did so least often. Those with a longer duration of residence and better self-rated German language proficiency more often answered the questionnaire exclusively in German. All group differences were statistically significant (*p* = 0.020 for gender, *p* < 0.001 for the other indicators) according to chi²-tests.

## Discussion

We evaluated the different recruitment efforts undertaken to motivate a sample of persons with selected citizenships living in Germany to take part in the survey GEDA Fokus and how these efforts accounted for differences in the sample composition throughout the recruitment process.

### Differences in outcome rates according to AAPOR

The overall response rate of 18.4% is higher than in the previous feasibility study with a response rate of 15.9% conducted in Berlin and Brandenburg among people with Croatian, Polish, Romanian, Syrian or Turkish citizenship [23, 24]. However, it is lower than the mean of the anticipated response rates per citizenship group (22.1%) as described in the study protocol [25]. Response rates differed remarkably between citizenship groups, and the group with Turkish citizenship remained the hardest to motivate for participation throughout all contact phases. A possible explanation for this might be that people with own or familial Turkish history of migration are amongst the most often surveyed migrant groups in Germany, possibly leading to a certain degree of survey fatigue [22], also reflected in the lowest final cooperation rate. In contrast, the highest cooperation rate was observed among participants with Syrian citizenship. This might be due to a high participation rate via self-administered survey modes; the group with Syrian citizenship in the majority came to Germany during the recent years, hence, these people might feel less survey fatigue [22]. Furthermore, the commitment of the interviewers, some of whom originated from Syria themselves, might have contributed. Working with interviewers from similar backgrounds and countries of origin is also recommended to motivate people for survey participation, because it builds trust and might bridge language gaps [12, 38]. Due to their high rate of participation via self-administered survey modes in the early and intermediate contact phases, the lowest contact rate was observed in the group with Italian citizenship. Only a few home visits were required to include the intended sample size of 1200 participants, resulting in many case closures of unknown eligibility and therefore a low contact rate. In contrast, the highest contact rate was observed in the group with Polish citizenship, with a sharp increase in the last contact phase. Interviewers made an effort motivating this group for survey participation during home visits, which also resulted in many refusals. However, after quality checks in terms of completeness of questionnaires, this remained the only group in which we failed to achieve the aimed for 1200 participants.

Differing results with regard to the BIK classification of the PSUs show and underline that home visits [39] are considered as gold standard for *hard to survey* groups [14, 18–21]. However, as home visits are expensive, telephone contacts might be an alternative [40] if telephone numbers can be obtained, which has become increasingly difficult in Germany in recent years [26]. But as offering self-administered survey modes, particularly the web-based one, is cheaper than engaging interviewers, either on the telephone or conducting home visits, the most cost-effective way is starting with self-administration.

### Development of the sample composition throughout recruitment

In the later stages of the recruitment process, more female and older participants were motivated to participate in the survey, as well as people with lower levels of education, income, German language proficiency, and persons born abroad. In terms of the impact of physical health indicators on recruitment there was a mixed picture. Those reporting physical health problems were recruited more often in the intermediate than the late contact phase. Nevertheless, participants in the later contact phases more often showed less favourable health behaviours. In contrast, intermediate and especially late participants less often reported symptoms of depression or anxiety as well as low social support, a lower sense of belonging to the society in Germany and experiences of discrimination. Against a background in which people with mental disorders are often underrepresented within surveys and show higher drop-out rates in longitudinal studies [41–44] this finding is surprising. However, as mental health and also psychosocial determinants such as social support, sense of belonging, or experiences of discrimination are sensitive topics, this finding might be explainable by mode effects leading to social desirability bias [45, 46], meaning that sensitive topics are more truthfully answered in self-administered modes, which were offered in the early contact phases in our study. We also observed that participants with symptoms of depression or anxiety and those with low social support, a lower sense of belonging, as well as experiences of discrimination, more often were among those self-administering the questionnaire, which supports this explanation. Such mode differences in mental health and psychosocial outcomes can also be found in the literature [47, 48]. However, differences in the sample composition among early, intermediate, and late participants might also explain these results. In the end, mental health researchers should be aware of such possible mode effects and possibly opt for offering self-administered survey modes. However, as the true overall prevalence of symptoms of depression or anxiety is unknown, we cannot tell if the prevalence among early or self-administering vs. late participants taking part in interviews comes closer to reality.

### Utilization of modes of survey administration

The sequential mixed-mode design, in combination with multiple contacts for initial non-responders, enabled us to include different subgroups of participants, as participants were able to choose their preferred mode throughout the recruitment process [38]. This is underpinned by the *Leverage-Salience Theory of Survey Participation* offered by Groves et al. [13], stating that different survey attributes may have varying cooperation effects on different subgroups of sample persons. Similar results on the utilization of modes of survey administration were found among foreign-born participants in Finland, using a comparable sequential mixed-mode design [49]. This underlines that every further contact attempt, but also additionally offered mode of survey administration, attracts and motivates further sample persons to participate in surveys. Offering saq-paper in addition to saq-web accounts for the phenomenon of ‘digital divide’, for example among older and younger participants [50]. Home visits that also offered the option to participate in an interview allowed the interviewers to better explain the aims of the study, hence including more participants with, for example, lower educational levels.

### Utilization of questionnaire language

Translations were used by nearly half of participants and especially from those with lower self-reported German language proficiency and shorter duration of residence. Hence, translations addressed language barriers for those who faced them. In addition, offering translations may have helped to establish trust and appreciation [8, 18] and thus increased the motivation for participation among all participants. In diversified societies it is essential to offer multilingual surveys in order to avoid the systematic exclusion of subgroups within the population [51], but also to acknowledge diversity. Offering translations of languages of the major migrant groups is necessary, accompanied by important link languages such as English, Russian, Spanish, or Arabic.

### Strengths and limitations

GEDA Fokus was the first large-scale health interview survey in Germany addressing specific migrant groups. Offering many different options to enable participation in combination with multiple contact attempts as well as offering an incentive allowed us to recruit a heterogeneous sample that will enable differentiated analyses on migration and health.

However, there are some limitations that need to be considered. As sampling was based on citizenship, we do not capture naturalized people with only German citizenship nor do we include people with other citizenships than those that were selected. Due to practical and financial constraints, for example costs for translations, we had to decide on five groups. Using model calculations as described in the study protocol [25], we tried to solve this issue in a comprehensible and replicable way. As sampling was furthermore based on residents’ registries, we also do not include those people who are not registered there, for example people without a legal immigration status. In sum, this leads to the fact that the generalizability of our survey results is limited to the sampled groups.

With 18.4% the realized response rate is relatively low, hence, there is probably a certain degree of nonresponse bias [52]. In times of constantly sinking response rates [7], however, a lot of surveys have to deal with this issue. Hence, we calculated weighting factors to account for that [25].

When analysing the sample composition throughout the recruitment process, we mostly need to rely on self-reported questionnaire data. Comparisons with the gross sample, and as such with the non-responders as well, are only possible in terms of very few register-based characteristics (gender, age, BIK classification). Other information, which was provided by the residents’ registration offices, is fragmentary – for example, on marital status (21.6% missing values) or on country of birth (16.1% missing values). Further information, such as educational attainment or occupational status, is not recorded in the registers at all [26]. Hence, non-responder analyses are hampered.

Comparisons between GEDA Fokus participants and participants of the same selected citizenships in interview surveys among the general population – as impressively presented by Galinsky et al. [16] for Native Hawaiians and Pacific Islanders in the United States – are not possible either, as these groups are not sufficiently represented in surveys of the general population. In addition, the differing study designs hamper such comparisons. Both analyses would have been desirable to assess the quality of our sample.

Another issue that may affect the data quality is the mixed-mode design itself. As mentioned, interviewer-administered survey modes may affect response behaviour, especially for sensitive topics due to social desirability bias [45]. This needs to be kept in mind and statistically controlled for, for example, when analysing mental health outcomes, psychosocial determinants of health, or other topics that participants may regard as sensitive.

### Conclusion and recommendations

Multiple contact attempts, including home visits and using multiple survey languages, as well as sequentially offering different modes of survey administration, are promising tools with which to raise response rates. Also, offering a conditional incentive might have impacted the willingness to participate. In addition, providing different options to participate may have helped to reduce survey response bias. During recruitment, we were able to include more participants of groups, that often remain non-responders, e.g. those with lower levels of education [53]. Implementing such a complex study design within national public health monitoring is costly. However, it might not only help to improve the inclusion of people with a history of migration, but also of other groups within the population considered *hard to survey* – for example, people living in poverty, single parents, or older individuals aged 65 or 80 years and above, respectively [54] – which is of further importance as the proportion of older people among the migrant population in Germany is constantly rising. National public health monitoring is obliged to represent a diversifying society as a whole. Thus, ‘*The ideal research study in a multicultural society would include all migrant*,* racial*,* and ethnic groups*,* have uniformly high response rates*,* provide data that are comparable across all groups (…)*’, as Bhopal [55] (p. 290) puts it; this ideal way of conducting research would therefore reduce sampling bias as well as non-response bias. In the light of already constantly decreasing response rates it is therefore essential to permanently develop survey methods further to suit the needs of all people within the society to facilitate their survey participation. Only this way is the identification of subgroups within the population that are especially affected by health inequities possible, which is essential to determine their specific health needs. This can serve as a basis for targeted evidence-based public health measures to sustainably improve health.

## Data Availability

The datasets generated and analysed during the current study are not publicly available due to data protection restrictions but are available from the corresponding author on reasonable request.

## Footnotes

1 Salentin and Schmeets (2017) [26] also suggest *country of birth* being reliably captured; however, our experiences differ. Therefore, citizenship was selected as the sampling criterion.

2 In Germany, it is possible to have dual or multiple citizenship e.g. for people with citizenships of the EU member states or Switzerland and under specific circumstances also for people with citizenships of other countries, e.g. when they cannot discard their “old” citizenship during the process of naturalization in Germany. But further possibilities also exist.

3 A detailed description of the professional translation approach as well as topics covered within the questionnaire can be found in the study protocol [25].

4 Core areas of cities with 100,000 to 500,000 inhabitants are ascribed a BIK classification of 8, classifications 9 and 10 are ascribed to the core and surrounding areas of cities with ≥ 500,000 inhabitants [28].

## Abbreviations

AAPOR: American Association for Public Opinion Research
BIK classification: Regional classification system for Germany provided by the BIK ASCHPURWIS + BEHRENS GmbH, Hamburg
CAPI: Computer-assisted personal interview
CATI: Computer-assisted telephone interview
CI: Confidence interval
EHIS-PAQ: European Health Interview Survey – Physical activity questionnaire
GAD-7: 7-item anxiety scale
GEDA: German Health Update (‘Gesundheit in Deutschland aktuell‘)
GEDA Fokus: German Health Update: Fokus (‘Gesundheit in Deutschland aktuell: Fokus‘)
I: Complete Interview (disposition code provided by AAPOR)
ISCED: International Standard Classification of Education
NC: Non-contact (disposition code provided by AAPOR)
NE: Not eligible (disposition code provided by AAPOR)
O: Other, non-refusals (disposition code provided by AAPOR)
OSSS-3: 3-item Oslo Social Support Scale
P: Partial interview (disposition code provided by AAPOR)
PHQ-9: 9-item version of the Patient Health Questionnaire
PSUs: Primary sampling units
R: Refusal and breakoff (disposition code provided by AAPOR)
RKI: Robert Koch Institute
saq-paper: Self-administered questionnaire, paper-based
saq-web: Self-administered questionnaire, web-based
UH: Unknown if household (disposition code provided by AAPOR)
UO: Unknown Other (disposition code provided by AAPOR)
WHO: World Health Organization

## References

[1] McAuliffe M, Triandafyllidou A. World Migration Report 2022. International Organization for Migration (IOM). Geneva, Switzerland; 2017.

[2] McAuliffe M, Ruhs M. World Migration Report 2018. International Organization for Migration (IOM). Geneva, Switzerland; 2017.

[3] Statistisches Bundesamt (Destatis). Bevölkerung und Erwerbstätigkeit. Bevölkerung mit Migrationshintergrund. Ergebnisse des Mikrozensus 2022. (Erstergebnisse). In: *Fachserie 1 Reihe 2.2*. Statistisches Bundesamt (Destatis): Wiesbaden, Germany; 2023.

[4] Statistisches Bundesamt (Destatis). Statistischer Bericht - Mikrozensus - Bevölkerung nach Einwanderungsgeschichte – Erstergebnisse 2022. Statistisches Bundesamt (Destatis): Wiesbaden, Germany; 2023.

[5] Kraler A, Reichel D. Statistics on migration, integration and discrimination in Europe. PROMISTAT Final Report. International Centre for Migration Policy Development: Vienna, Austria; 2010.

[6] Feskens RCW. Difficult groups in research and the development of tailor-made approach strategies. University of Utrecht: Utrecht, Netherlands; 2009.

[7] Bacher J, Lemcke J, Quatember A, Schmich P. Probability and nonprobability sampling: representative surveys of hard-to-reach and hard-to-ask populations. Current surveys between the poles of theory and practice. Survey Methods: Insights from the Field. 2019. https://surveyinsights.org/?p=12070. Accessed 31 Jan 2024.

[8] Koschollek C, Kajikhina K, Bartig S, Zeisler ML, Schmich P, Gößwald A, et al. Results and strategies for a diversity-oriented public health monitoring in Germany. Int J Environ Res Public Health. 2022;19(2):798. 35055619 10.3390/ijerph19020798 PMC8775825

[9] Vonneilich N, Becher H, Bohn B, Brandes B, Castell S, Deckert A, et al. Associations of migration, socioeconomic position and social relations with depressive symptoms – analyses of the German National Cohort Baseline Data. Int J Public Health. 2023;68:1606097. 37533684 10.3389/ijph.2023.1606097 PMC10391163

[10] Bartig S, Beese F, Wachtler B, Grabka MM, Mercuri E, Schmid L, et al. Socioeconomic differences in SARS-CoV-2 infection and vaccination in Germany: a seroepidemiological study after one year of COVID-19 vaccination campaign. Int J Public Health. 2023;68:1606152. 37780135 10.3389/ijph.2023.1606152 PMC10538434

[11] GESIS – Lebnitz Institut für Sozialwissenschaften. ZA5280 Datenfile Version 2.0.0. GESIS – Lebnitz Institut für Sozialwissenschaften: Cologne, Germany; 2023.

[12] Tourangeau R. Defining hard-to-survey populations. In: Tourangeau R, et al. editors. Hard-to-survey populations. Cambridge, UK: Cambridge University Press; 2014.

[13] Groves RM, Singer E, Corning A. Leverage-saliency theory of survey participation: description and an illustration. Public Opin Q. 2000;64(3):299–308. 11114270 10.1086/317990

[14] Stoop I. Representing the populations: what general social surveys can learn from surveys among specific groups. In: Tourangeau R, et al. editors. Hard-to-survey populations. Cambridge, UK: Cambridge University Press; 2014.

[15] Castaneda AE, Rask S, Härkänen T, Juntunen T, Skogberg N, Mölsä M, et al. Enhancing survey participation among foreign-born populations: experiences from the Finnish migrant health and wellbeing study. (Maamu) Finnish Yearbook of Population Research. 2019;53:89–110. 10.23979/fypr.74048

[16] Galinsky AM, Simile C, Zelaya CE, Norris T, Panapasa SV. Surveying strategies for hard-to-survey populations: lessons from the native hawaiian and Pacific Islander National Health Interview survey. Am J Public Health. 2019;109(10):1384–91. 31415207 10.2105/AJPH.2019.305217 PMC6727300

[17] WHO Regional Office for Europe. Participatory health research with migrants: a country implementation guide. WHO Regional Office for Europe: Copenhagen, Denmark; 2022.

[18] Bonevski B, Randell M, Paul C, Chapman K, Twyman L, Bryant J et al. Reaching the hard-to-reach: a systematic review of strategies for improving health and medical research with socially disadvantaged groups. BMC Med Res Methodol. 2014:1445). 10.1186/1471-2288-14-42 PMC3974746 24669751

[19] Méndez M, Ferreras M, Cuesta M. Immigration and general population surveys in Spain: the CIS surveys. In: Font J, Méndez M, editors. Surveying ethnic minorities and immigrant populations. Methodological challenges and research strategies. Amsterdam, Netherlands: Amsterdam University Press; 2013. pp. 195–218.

[20] Brücker H, Rother N, Schupp J, IAB-BAMF-SOEP-Befragung von. Geflüchteten 2016: Studiendesign, Feldergebnisse sowie Analysen zu schulischer wie beruflicher Qualifikation, Sprachkenntnissen sowie kognitiven Potenzialen. In: Politikberatung kompakt. DIW Berlin; 2018.

[21] Duque I, Ballano C, Pérez C. The 2007 Spanish national immigrant survey (ENI1): sampling from the Padrón. In: Font J, Méndez M, editors. Surveying ethnic minorities and immigrant populations. Methodological challenges and research strategies. Amsterdam, Netherlands: Amsterdam University Press; 2013. pp. 69–80.

[22] Borgmann LS, Waldhauer J, Bug M, Lampert T, Santos-Hövener C. Zugangswege zu Menschen Mit Migrationshintergrund für die epidemiologische Forschung - Eine Befragung Von Expertinnen Und Experten. Bundesgesundheitsblatt - Gesundheitsforschung - Gesundheitsschutz. 2019;62:1397–405. 31650187 10.1007/s00103-019-03036-3

[23] Zeisler ML, Bilgic L, Schumann M, Wengler A, Lemcke J, Gößwald A, et al. Interventions to increase the reachability of migrants in Germany with health interview surveys: mixed-mode feasibility study. JMIR Form Res. 2020;4(4):e14747. 32293576 10.2196/14747 PMC7191348

[24] Zeisler ML, Lemcke J, Bilgic L, Lampert T, Santos-Hövener C, Schmich P. Integration of migrant populations into health monitoring in Germany: results from a feasibility study. Survey Methods: Insights from the Field, 2019. https://surveyinsights.org/?p=10780. Accessed 31 Jan 2024.

[25] Koschollek C, Zeisler ML, Houben RA, Geerlings J, Kajikhina K, Bug M, et al. German health update Fokus (GEDA Fokus) among residents with Croatian, Italian, Polish, Syrian, or Turkish citizenship in Germany: protocol for a multilingual mixed-mode interview survey. JMIR Res Prot. 2023;12:e43503. 10.2196/43503 PMC10134013 36790192

[26] Salentin K, Schmeets H. Sampling immigrants in the Netherlands and Germany. Comp Migr Stud. 2017;5(21). 10.1186/s40878-017-0062-2 PMC5730621 29264233

[27] American Association for Public Opinion Research (AAPOR). Standard definitions, final dispositions of case codes and outcome rates for surveys. 9th ed. American Association for Public Opinion Research (AAPOR): Alexandria, VA, USA; 2016.

[28] Behrens K, Böltken F, Dittmar H, et al. Regionale Standards: Ausgabe 2019. Arbeitsgruppe Regionale Standards. GESIS – Leibniz-Institut für Sozialwissenschaften: Cologne, Germany; 2019.

[29] Eurostat. Internationale Standardklassifikation für das Bildungswesen (ISCED). 2017. https://ec.europa.eu/eurostat/statistics-explained/index.php?title=Glossary:International_standard_classification_of_education_(ISCED)/de#:~:text=Die%20Internationale%20Standardklassifikation%20f%C3%BCr%20das,Daten%20ab%202014%20ISCED%202011. Accessed 31 Jan 2024.

[30] OECD. What are equivalence scales? OECD Publishing: Paris, France; 2011.

[31] Eurostat. Glossary: minimum European health module (MEHM). 2017. https://ec.europa.eu/eurostat/statistics-explained/index.php?title=Glossary:Minimum_European_Health_Module_(MEHM). Accessed: 31 Jan 2024.

[32] Finger JD, Tafforeau J, Gisle L, Oja L, Ziese T, Thelen J, et al. Development of the European health interview survey – physical activity questionnaire (EHIS-PAQ) to monitor physical activity in the European Union. Arch Public Health. 2015;73:59. 26634120 10.1186/s13690-015-0110-z PMC4667448

[33] Kroenke K, Spitzer RL, Williams JBW. The PHQ-9. Validity of a brief depression severity measure. J Gen Intern Med. 2001;16:606–13. 11556941 10.1046/j.1525-1497.2001.016009606.x PMC1495268

[34] Manea L, Gilbody S, McMillan D. Optimal cut-off score for diagnosing depression with the Patient Health Questionnaire (PHQ-9): a meta-analysis. CMAJ. 2012;184(3):E191–6. 22184363 10.1503/cmaj.110829 PMC3281183

[35] Spitzer RL, Kroenke K, Williams JBW, Löwe B. A brief measure for assessing generalized anxiety disorder. The GAD-7. Arch Intern Med. 2006;166(10):1092–7. 16717171 10.1001/archinte.166.10.1092

[36] Meltzer H. Development of a common instrument for mental health. In: Nosikov A, Gudex C, editors. EUROHIS developing common instruments for health surveys. Amsterdam, Netherlands: IOS Press; 2003. pp. 21–34.

[37] Schumann M, Kajikhina K, Polizzi A, Sarma N, Hoebel J, Bug M, et al. Concepts for migration-sensitive health monitoring. J Health Monit. 2019;18(3):49–65. 10.25646/6075 PMC8734173 35146253

[38] Haan M, Ongena Y. Tailored and targeted designs for hard-to-survey populations. In: Tourangeau R, et al. editors. Hard-to-survey populations. Cambridge, UK: Cambridge University Press; 2014. pp. 555–74.

[39] Kappelhof JWS. The effect of different survey designs on nonreponse in surveys among non-western minorities in the Netherlands. Surv Res. 2013;8(2):81–98.

[40] West BT, Zhang S, Wagner J, Gatward R, Saw H-W, Axinn WG. Methods for improving participation rates in national self-administered web/ mail surveys: evidence from the United States. PLoS ONE. 2023;18(8):e0289695. 37540678 10.1371/journal.pone.0289695 PMC10403122

[41] Allott K, Chanen A, Yuen HP. Attrition bias in longitudinal research involving adolescent psychiatric outpatients. J Nerv Ment. 2006;194(12):958–61. 10.1097/01.nmd.0000243761.52104.91 17164636

[42] Haapea M, Miettunen J, Läärä E, Joukamaa MI, Järvelin MR, Isohanni MK, Veijola JM. Non-participation in a field survey with respect to psychiatric disorders. Scand J Public Health. 2008;36(7):328–36. 10.1177/1403494808092250 18647788

[43] Perez FP, Baffour B. Respondent mental health, mental disorders and survey interview outcomes. Surv Res Meth. 2018;12(8):161–76.

[44] Momen NC, Lasgaard M, Weye N, Edwards J, McGrath JJ, Plana-Ripoll O. Representativeness of survey participants in relation to mental disorders: a linkage between national registers and a population-representative survey. Int J Popul Data Sci. 2022;7(4):1759. 37152406 10.23889/ijpds.v7i4.1759 PMC10161967

[45] Tourangeau R, Yan T. Sensitive questions in surveys. Psychol Bull. 2007;133(5):859–83. 17723033 10.1037/0033-2909.133.5.859

[46] Schork J, Riillo CAF, Neumayr J. Survey mode effects on objective and subjective questions: evidence from the Labour Force Survey. J off Stat. 2021;37(1):213–37. 10.2478/jos-2021-0009

[47] Zhang XC, Kuchinke L, Woud ML, Velten J, Margraf J. Survey method matters: online/offline questionnaires and face-to-face or telephone interviews differ. Comput Hum Behav. 2017;71:172–80. 10.1016/j.chb.2017.02.006

[48] Piccitto G, Liefbroer AC, Emery T. Does the survey mode affect the association between subjective well-being and its determinants? An experimental comparison between face-to-face and web mode. J Happiness Stud. 2022;23:3441–61. 10.1007/s10902-022-00553-y

[49] Kuusio H, Seppänen A, Somersalo L, Jokela S, Castaneda AE, Abdulhamed R, Lilja E. Response activity in mixed-mode survey data collection: the methods used in a survey among the foreign-born population in Finland (FinMonik). Int J Environ Res Public Health. 2021;18(6):3300. 33806759 10.3390/ijerph18063300 PMC8005148

[50] Mauz E, Hoffmann R, Houben RA, Krause L, Kamtsiuris P, Gößwald A. Mode equivalence of health indicators between data collection modes and mixed-mode survey designs in population-based health interview surveys for children and adolescents: methodological study. J Med Internet Res. 2018;20(3):e64. 29506967 10.2196/jmir.7802 PMC5859740

[51] Méndez M, Font J. Surveying immigrant populations: methodological strategies, good practices and open questions. In: Font J, Méndez M, editors. Surveying ethnic minorities and immigrant populations. Methodological challenges and research strategies. Amsterdam, Netherlands: Amsterdam University Press; 2013. pp. 271–90.

[52] Groves RM. Nonresponse rates and nonresponse bias in household surveys. Public Opin Q. 2006;70(5):646–75. 10.1093/poq/nfl033

[53] Koponen P, Aromaa A. Survey design and methodology in national health interview and examination surveys. Review of literature, European survey experiences and recommendations. Helsinki, Finland: National Public Health Institute (KTL); 2000.

[54] Gaertner B, Lüdtke D, Koschollek C, Grube MM, Baumert J, Scheidt-Nave C et al. Effects of a sequential mixed-mode design on participation, contact and sample composition: results of the pilot study IMOA – Improving Health Monitoring in Old Age. Survey Methods: Insights from the Field. 2019. https://surveyinsights.org/?p=10841. Accessed 31 Jan 2024.

[55] Bhopal R. Migration, ethnicity, race, and health in multicultural societies. Oxford, UK: Oxford University Press; 2014.

